# Practical consensus recommendations for polytherapy involving stiripentol in Dravet syndrome: A nominal group approach

**DOI:** 10.1002/epi4.70245

**Published:** 2026-03-18

**Authors:** J. Helen Cross, Rima Nabbout, Barry Gidal, Joseph Sullivan, Elaine Wirrell

**Affiliations:** ^1^ UCL‐NIHR BRC Great Ormond Street Institute of Child Health Great Ormond Street Hospital for Children NHS Foundation Trust London UK; ^2^ Member of European Reference Network EpiCARE, Department of Pediatric Neurology, Reference Centre for Rare Epilepsies, Hôpital Necker‐Enfants Malades, Assistance Publique Hôpitaux de Paris, Institut Imagine, U1163 Paris cité University Paris France; ^3^ School of Pharmacy University of Wisconsin‐Madison Madison Wisconsin USA; ^4^ Department of Neurology and Pediatrics, Benioff Children's Hospital University of California San Francisco California USA; ^5^ Division of Child and Adolescent Neurology and Epilepsy, Department of Neurology Mayo Clinic Rochester Minnesota USA

**Keywords:** antiseizure medications, combination therapy, Dravet syndrome, expert consensus, nominal group technique, stiripentol

## Abstract

**Objectives:**

Dravet syndrome (DS) is a drug‐resistant developmental and epileptic encephalopathy, often requiring three or more antiseizure medications (ASMs). Consequently, the therapeutic landscape is highly complex, combining DS‐specific agents (stiripentol, STP; cannabidiol, CBD; fenfluramine, FFA), non‐specific ASMs, and non‐pharmacologic options such as dietary therapy or neuromodulation. In addition, recognition of patients' changing needs across the lifespan, together with the essential contribution of caregiver education to effective treatment oversight—including awareness of potential drug–drug interactions and adverse events—emphasizes the value of an integrated and forward‐looking approach. A consensus initiative was developed to guide clinicians in the management of patients with DS, with a particular focus on STP‐containing regimens.

**Methods:**

Based on a literature review and clinical vignette discussions, a nominal group of five international DS experts developed 38 statements across six themes: (i) general principles, (ii) guiding physicians to empower families, (iii) STP as add‐on to specific and non‐specific Dravet ASMs, (iv) FFA in regimens containing STP, (v) CBD in regimens containing STP, and (vi) patient follow‐up during treatment initiation. Statements were scored on a 9‐point Likert scale (1 = strongly disagree, 9 = strongly agree) first by the nominal group and then by an international panel of 59 DS clinicians. Consensus was defined as ≥75% rating ≥7 and/or a median ≥8.

**Results:**

Thirty‐four of the 38 statements (89%) reached consensus among both the nominal group and the international panel; one did not reach consensus in either, and three reached consensus only among the nominal group. Both panels agreed on the importance of involving families in treatment decisions and follow‐up, as well as on key aspects of polytherapy management, including ASM initiation, treatment adjustments, and adverse event management.

**Significance:**

This international consensus integrates evidence and clinical expertise to establish shared principles that address a long‐standing gap in the polytherapeutic management of Dravet syndrome.

**Plain Language Summary:**

Dravet syndrome is a rare and severe form of epilepsy that begins in early childhood, with seizures that often persist throughout life. Because the disease is highly drug resistant, most patients are treated with polytherapy, making clinical management complex for clinicians. This consensus brings together published evidence and expert experience to provide practical guidance on the management of polytherapy in Dravet syndrome, supporting clinicians in optimizing patient care.

**Key points**

- Dravet syndrome is a rare developmental and epileptic encephalopathy characterized by drug‐resistant, lifelong seizures and comorbidities.
- The therapeutic landscape is diverse, complex, and changing throughout the life span, adding to the challenges of optimizing patient care.
- This international consensus integrates literature evidence and clinical expertise to define practical principles of polytherapy management beyond individualized care to support pediatric and adult neurologists in optimizing DS management.

## 1 INTRODUCTION

Dravet syndrome (DS) is a rare and severe drug resistant developmental epileptic encephalopathy that starts in infancy, presenting with seizures—most often bilateral tonic–clonic—which are frequently prolonged and triggered by fever or minor infections. With age, additional seizure types emerge, and children often develop cognitive impairment, behavioral disturbances, sleep disorders, and motor difficulties, creating a complex clinical picture that significantly reduces quality of life for both patients and their caregivers, with wide‐ranging emotional, physical, and social consequences.^1^, ^2^, ^3^, ^4^, ^5^

The clinical management of DS involves optimizing antiseizure medications (ASMs) and addressing co‐morbidities with tailored support for both patients and caregivers. Given the drug‐resistant nature of this disease, polytherapy is the standard of care, with a vast majority of patients receiving three ASMs concurrently.^6^, ^7^, ^8^ Clinical guidelines, supported by several publications,^6^, ^9^, ^10^, ^11^ generally recommend valproate (VPA)—and clobazam (CLB) in some regions—as first‐line options despite limited evidence of efficacy,^11^, ^12^ which likely reflects the intrinsic drug‐resistant nature of the condition. Second‐line therapies, used as adjuncts to VPA and/or CLB, comprise the three agents specifically approved for DS, each supported by robust evidence from randomized controlled trials:

1. Stiripentol (STP), the first DS‐specific drug authorized in Europe (2007), in conjunction with VPA and CLB with no age restriction, and in the US (2018), in conjunction with CLB for children aged ≥6 months and weighing ≥7 kg,^13^, ^14^
2. Fenfluramine (FFA, authorized in Europe and in the US in 2020 for children 2 years and older),^15^
3. Cannabidiol (CBD, authorized in the US in 2018 for children 1 year and older and in Europe in 2019 in conjunction with CLB for patients 2 years and older).^16^

Among these three drugs, the most recent recommendations consider STP and FFA as second‐line options, with CBD considered as a third‐line option.^6^, ^11^ Other ASMs such as topiramate, levetiracetam, or bromides may be considered in selected cases, although the evidence for efficacy in DS remains limited.^12^ Non‐pharmacologic approaches such as dietary therapy (ketogenic diet) or neuromodulation may also offer benefits.^6^ Considering the early onset of the disorder and the critical importance of initiating treatment promptly to reduce seizure frequency and duration and potentially limit neurological deterioration,^17^ STP remains the only DS‐specific ASM that can be prescribed early on (i.e., in patients younger than 1 year). Overall, the choice of combination therapy is highly dependent on the age of the patient, local drug availability, country‐specific recommendations, and the clinical judgment of the treating healthcare professional (HCP).^6^, ^18^, ^19^

The number of available treatments, combined with the heterogeneity of seizure types,^17^ the frequent reliance on polytherapy, and the high rate of comorbidities create a highly complex therapeutic landscape for clinicians.^11^, ^20^ Before adding a new treatment to an existing ASM regimen, multiple factors must be carefully considered, including efficacy, potential drug–drug interactions, pharmacokinetic profiles, pediatric‐friendly formulations, adverse effects management, potential impact on comorbidities, and the need for blood monitoring.^21^ In parallel, the evolving needs of patients and families throughout the Dravet journey further emphasize the holistic complexity of optimizing DS management.^22^

The aim of this work was to support HCPs in optimizing treatment combinations for patients with Dravet syndrome. Given the early onset of seizures, the importance of initiating Dravet syndrome‐specific treatments as early as possible, and the fact that STP is currently the only DS‐specific antiseizure medication authorized for use at a very young age, with no age restriction in Europe, a particular focus was placed on regimens involving STP. To this end, a modified nominal group technique (NGT) was used to reach expert consensus and develop clear and precise practical guidance.

## 2 MATERIALS AND METHODS

### 2.1 Nominal group technique

The NGT is a structured and interactive method used to generate both knowledge in an exploratory approach and to achieve consensus among a panel of experts on a clearly defined issue. It encourages equal participation from all members, ensuring that every idea is considered in the decision‐making process.^23^, ^24^, ^25^, ^26^ This method of consensus building has been applied in various healthcare fields, including priority setting in research, medical education, physiotherapy, and evaluation of medical practices in various domains (rheumatology, cardiology, pulmonology, pediatrics, etc.).^23^, ^27^, ^28^

In our study, experts were selected for their recognized expertise in Developmental and Epileptic Encephalopathy (DEE) and drug interaction management. The final group comprised four pediatric neurologists (two from the United States, one from France, and one from the United Kingdom) and one clinical pharmacologist (from the United States), all of whom have extensive clinical experience in the care of patients with DS, have authored numerous publications in the field, and are actively involved in international expert boards and patient advocacy group scientific committees.

A comprehensive literature review was conducted by the steering committee prior to the first meeting to provide a scientific basis for discussing the optimization of treatment combinations in DS, with a particular focus on regimens involving STP. Relevant data on pharmacological management, polytherapy, and drug–drug interactions were identified through a systematic PubMed search using the keywords: “Dravet syndrome,” “polytherapy,” “stiripentol,” “treatment combinations,” and/or “drug interactions.” The search period was restricted to 2018 onward, corresponding to the approval of CBD and FFA, which significantly reshaped the therapeutic landscape. Of the 60 publications identified, 15 peer‐reviewed articles were retained to ensure comprehensive coverage of therapeutic associations and pharmacological interactions relevant to DS, based on their contribution to the current understanding of the disease, its management, and the pharmacokinetic and pharmacodynamic interactions of ASM used in DS patients.

The first NGT experts meeting was held virtually in January 2025 as a 3‐h group video conference and structured as follows.

#### 2.1.1 Individual silent analysis

To structure discussions, five practical clinical cases inspired by real‐world scenarios were used for expert analysis (Figures S1–S5).^29^ These clinical scenarios provided a framework to organize key considerations commonly addressed when adding a new treatment to an existing regimen, in order to facilitate the development of practical recommendations and ensure that the statements submitted for voting reflected real‐world clinical practice:

1. Patient treated with a regimen containing non‐specific ASMs for whom addition of STP is being considered.
2. Patient treated with a regimen containing FFA, for whom the addition of STP is being considered.
3. Patient treated with a regimen containing CBD, for whom the addition of STP is being considered.
4. Patient treated with a regimen containing STP, for whom the addition of FFA is being considered.
5. Patient treated with a regimen containing STP, for whom the addition of CBD is being considered.

For each clinical situation, seven standardized questions were posed, addressing key aspects of the new treatment initiation. Although organized to mirror a typical clinical pathway, all questions were presented simultaneously. Thus, each expert was able to provide anonymous written responses on (i) expected benefits of adding a new drug to a previous regimen; (ii) management of the drug introduction; (iii) management of other drugs in the regimen; (iv) potential adverse events due to the addition of the new drug and their management; (v) new monitoring and organization of follow‐up; (vi) communication with parents; and (vii) any other relevant comments.

#### 2.1.2 Group discussion

A group discussion followed, allowing experts to exchange and debate their individual responses. They were encouraged to consolidate existing ideas and propose new ones. From these discussions, participants developed clinical practice recommendations, organized by category and tailored to address specific aspects of managing the addition of a new treatment in DS patients.

#### 2.1.3 Voting process

One week after the nominal group meeting, and in accordance with NGT‐specific adaptations,^26^ experts proceeded to rate the generated recommendation statements using a nine‐point Likert scale (1 = strongly disagree, 9 = strongly agree) via a personalized link to an online survey platform. Consensus thresholds were defined as follows:^30^

1. A “strong consensus” was reached if more than 75% of scores were ≥7 and the median score was ≥8.
2. A “good consensus” was considered if only one of these two criteria was met.
3. “No consensus” was determined if neither criterion was met.

The pharmacologist expert, who does not have direct clinical involvement in the management of patients with DS, chose to abstain from voting.

### 2.2 Extended vote to a larger number of DS specialists

**FIGURE 1:**
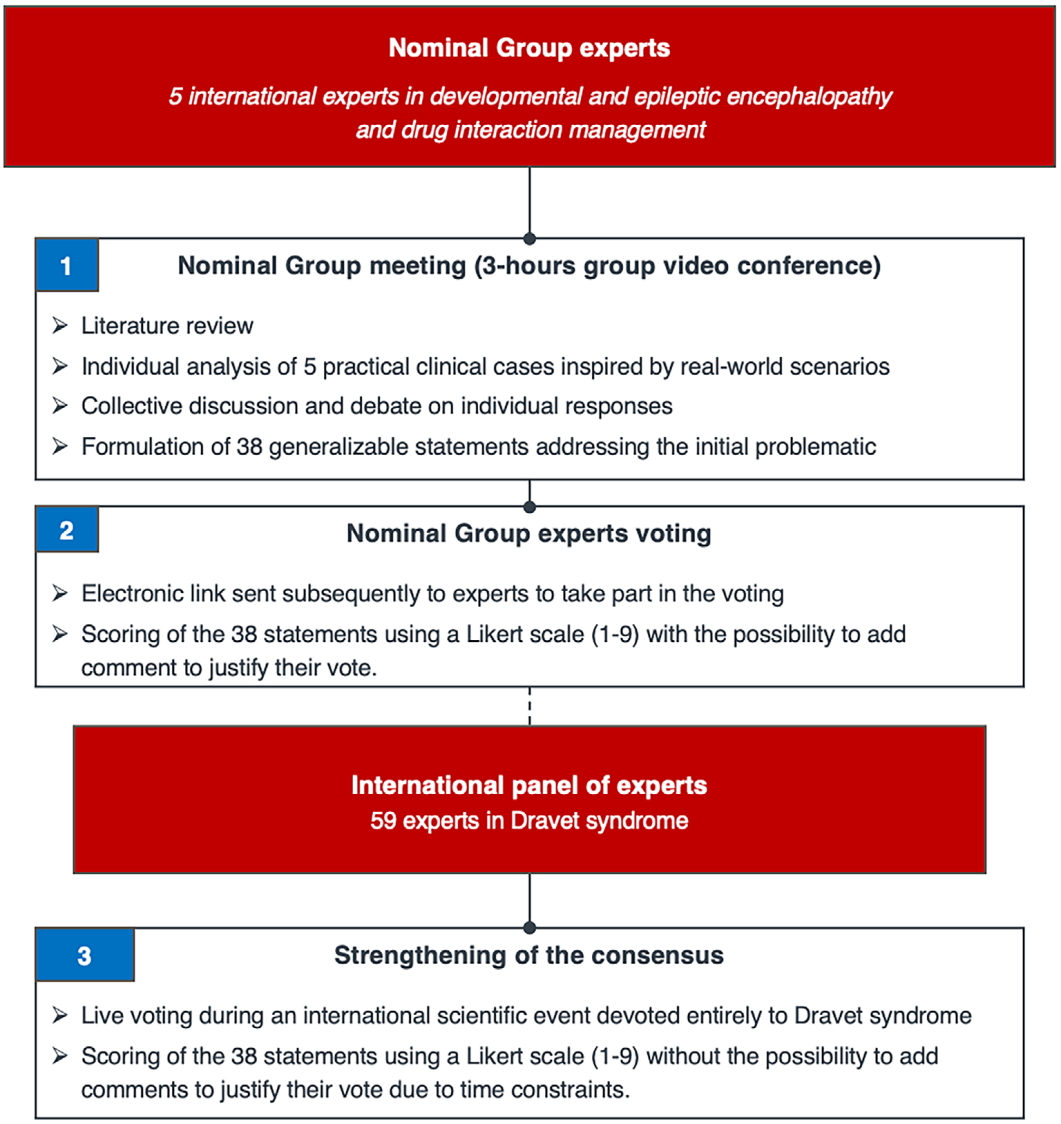
Modified group nominal procedure used in our study.

**FIGURE 2:**
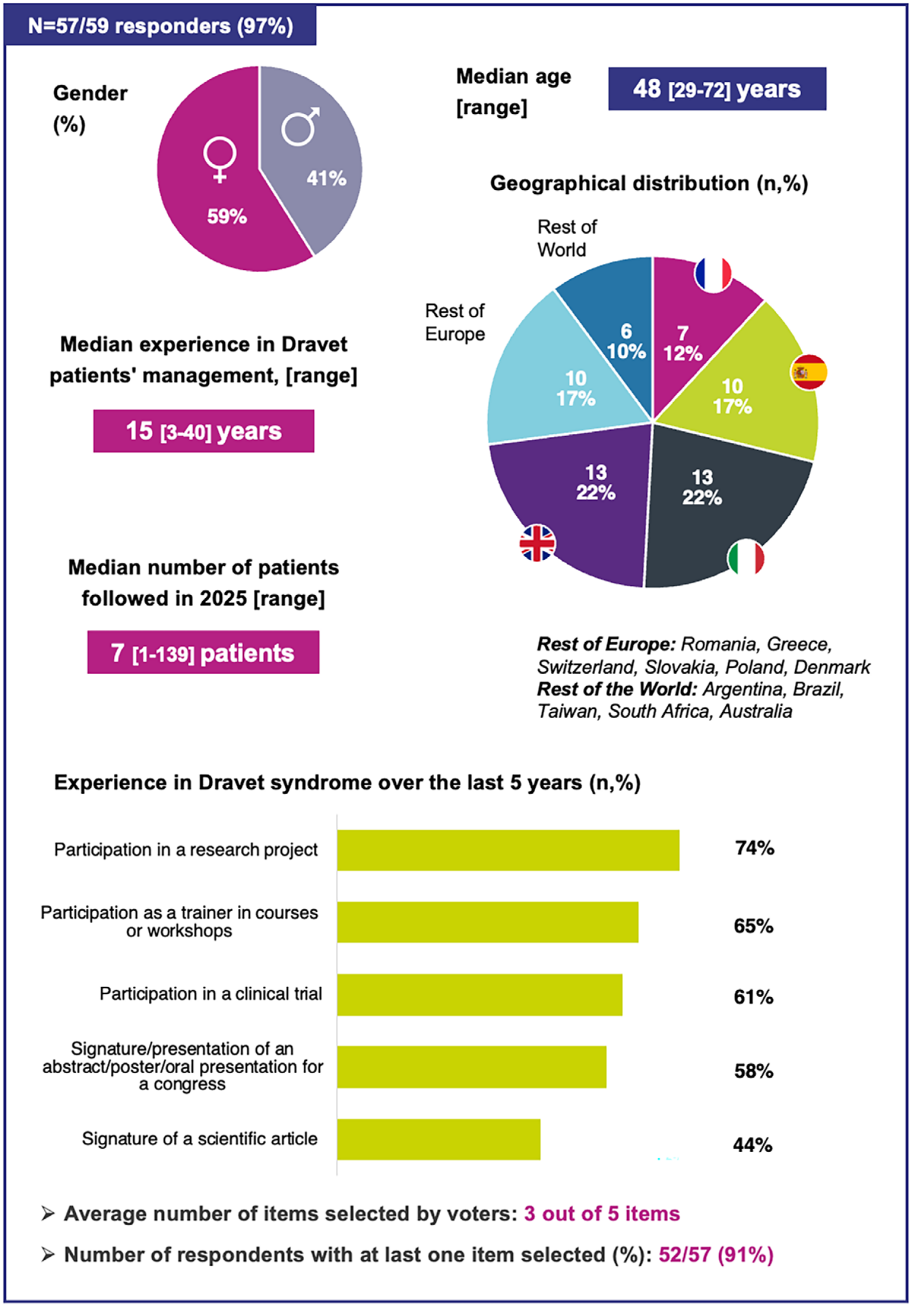
Characteristics of the international voting panel.

Following the vote of the nominal group experts, the consensus process was reinforced by a broader panel of 59 DS specialists, mostly from Europe, who voted during an international DS scientific event, using the same consensus methodology as the nominal group vote (Figure 1). Figure 2 summarizes participants' characteristics and experience in DS, with 57 of the 59 (97%) completing the characterization survey. The cohort showed disparities in the number of DS patients currently followed, with a median of 7 [1–139] patients. However, overall experience in the field was well established, with a median of 15 [3–40] years since first managing patients with DS and 91% of voters (52/57) having been involved in at least one DS‐related activity over the past 5 years, including participation in a DS research project (74%), training in DS courses or workshops (65%), participation in a DS clinical trial (61%), presentation at a DS congress (58%), or authorship of a scientific publication on DS (44%).

The questionnaire format and rating scale were identical to those used with the nominal group. Voting was conducted without additional comments due to time constraints.

Finally, in June 2025, a follow‐up meeting with the nominal group experts was convened to review, discuss, and summarize the final results.

## 3 RESULTS

During the nominal group process, experts formulated 38 statements, organized into six sections: (i) General principles, (ii) Guiding physician to empower patients, (iii) STP as an add‐on to specific and non‐specific Dravet ASMs, (iv) FFA in a regimen containing STP, (v) CBD in a regimen containing STP, and (vi) Patient follow‐up during treatment initiation (Table 1).

**TABLE 1:** Voting results of the nominal group experts and the international expert panel.

	Statements	Nominal group experts (*n* = 4)	International experts panel (*n* = 59)
**General principles**			
1	Given the propensity for prolonged seizures in Dravet Syndrome, every patient should be prescribed a rescue medication, and a clear seizure action plan should be provided	Strong consensus	Strong consensus
2	Before considering a switch or introducing a new medication, priority should be given to optimizing the dosage of an existing treatment, taking into consideration efficacy and tolerability.	Strong consensus	Strong consensus
3	When a new treatment is introduced and adjustments are made to the ASM regimen, a change in seizure frequency and/or severity may occur. A sufficient time should be given to evaluate the effectiveness (efficacy and tolerability) of the new treatment.	Strong consensus	Strong consensus
4	Sodium Channel Blocking ASM should be avoided following a diagnosis of Dravet Syndrome.	Strong consensus	Strong consensus
5	When adding STP or CBD to any base regimen, potential adverse effects can usually be assessed clinically, and plasma levels are not mandatory to optimize treatment effectiveness (efficacy and tolerability).	Strong consensus	Good consensus
6	ASM changes may partly impact comorbidities; however, the underlying causes of comorbidities are multifactorial. Comorbidities require ongoing evaluation and targeted intervention.	Strong consensus	Strong consensus
7	Following the introduction of STP, if an existing ASM did not afford significant seizure improvement, then tapering it off over 4–8 weeks should be considered.	Strong consensus	No consensus
**Guiding the physician to empower parents or caregivers**			
8	Physicians should actively involve parents or caregivers in treatment decisions and discuss the advantages and disadvantages of the various available options, including ASM, dietary therapies, and neuromodulation.	Strong consensus	Strong consensus
9	Physicians should discuss with parents or caregivers about referral and potential inclusion in clinical trials.	Strong consensus	Strong consensus
10	When initiating a new treatment, the physician should give parents or caregivers clear expectations regarding treatment efficacy and possible adverse effects.	Strong consensus	Strong consensus
11	Physicians should provide parents or caregivers with a clear plan, including written information regarding medication titration, tapering, as well as guidance on monitoring seizures and adverse effects, and when and how to seek medical advice.	Strong consensus	Strong consensus
**STP as an add‐on to other ASMs**			
12	When adding STP to any base regimen of ASM, including VPA and/or CLB, a significant reduction in seizure frequency and duration, as well as a significant decrease or cessation in episodes of prolonged convulsive seizures and status epilepticus, is generally anticipated within 4 to 6 weeks after reaching the target dose.	Strong consensus	No consensus
13	STP should be introduced to a regimen containing VPA and/or CLB.	Strong consensus	Strong consensus
14	When adding STP to any base regimen of ASM, initiation at 15–20 mg/kg/day is recommended, with a gradual increase over 2–4 weeks to the initial target dose, adjusted based on seizure frequency, efficacy, and tolerability.	Strong consensus	Strong consensus
15	When adding STP to any base regimen of ASM, the weight and age of the patient must be considered: children younger than 5 years old usually require a higher targeted dose (50 mg/kg/day), whereas adolescents require a lower targeted dose (30 mg/kg/day).	Strong consensus	Strong consensus
16	When adding STP to a base regimen containing VPA and CLB, early reduction of co‐medications at the initiation of STP should be considered to help minimize the occurrence of adverse effects, as they are most likely due to higher levels of those 2 co‐medications.	Strong consensus	Strong consensus
17	Considering drug interactions with CLB, when STP is introduced: If the regimen already contains CLB, adjusting CLB to a maximum 0.5 mg/kg/day should be considered. If adverse effects such as sedation, somnolence, ataxia are observed, tolerability can be improved by decreasing CLB by 25%.	Strong consensus	Strong consensus
18	Considering drug interactions with VPA, when STP is introduced: If the regimen already contains VPA, adjusting VPA to a maximum of 30 mg/kg/day should be considered. If adverse effects such as sedation, decreased appetite, weight loss, or thrombocytopenia are observed, tolerability can be improved by decreasing VPA by 25%.	Strong consensus	Strong consensus
19	Considering drug interactions with FFA, when STP is introduced, FFA must be adjusted to a maximum of 0.4 mg/kg/day, not exceeding 17 mg/day.	Strong consensus	Strong consensus
20	Considering drug interactions with CBD, when STP is introduced: No specific dose adjustment of CBD nor STP is required. The addition of STP is not expected to further induce pharmacokinetic interactions with concomitant co‐medications, such as CLB. Therefore, no further adjustment of CLB is required; however, careful clinical monitoring is recommended	Strong consensus	Strong consensus
21	Considering STP drug interactions with TPM or LEV, no specific dose adjustment is required.	Strong consensus	Strong consensus
**FFA in a regimen containing STP**			
22	When adding FFA to any base regimen of ASM, including STP, a significant reduction in seizure frequency is generally anticipated within 4 to 6 weeks after reaching the target dose.	Strong consensus	Strong consensus
23	When adding FFA to any base regimen of ASM, including STP, an initial dose of 0.1–0.2 mg/kg/day is recommended, with gradual titration by 0.1 mg/kg/day every 1–2 weeks based on treatment response and tolerability, aiming for a maximum dose of 0.4 mg/kg/day, not exceeding 17 mg/day.	Strong consensus	Strong consensus
24	Before introducing FFA to any base regimen, an echocardiography must be performed to confirm the absence of aortic or mitral valvular heart disease and pulmonary arterial hypertension.	Strong consensus	Strong consensus
25	When adding FFA to a regimen containing STP, VPA, and CLB, if a partial seizure improvement and/or SE reduction was observed following STP addition and prior to FFA initiation, maintaining STP should be considered.	Good consensus	Strong consensus
26	When adding FFA to a base regimen containing STP, VPA, and CLB, common adverse effects include decreased appetite, fatigue, and somnolence. These are often transient, but if present, a lower FFA target dose and slower FFA titration could be considered.	Good consensus	Good consensus
**CBD in a regimen containing STP**			
27	When adding CBD to any base regimen of ASM, including STP, a moderate reduction in overall seizure frequency is generally anticipated.	No consensus	No consensus
28	When adding CBD to any base regimen of ASM, including STP, initiation at 2.5 mg/kg/day is recommended, with 2 mg/kg/day weekly increases to an initial target dose of 10–12 mg/kg/day over 3–4 weeks. Treatment response should then be assessed, with further increases of 2.5 mg/kg/day every 1–2 weeks if seizures persist, up to a maximum dose of 20 mg/kg/day if well tolerated.	Strong consensus	Strong consensus
29	Since STP already has a pharmacokinetic interaction with CLB, no further dose reduction of CLB is necessary when adding CBD to a patient already receiving STP and CLB.	Strong consensus	No consensus
30	When adding CBD to any base regimen of ASM, including STP, the benefit of CBD should be assessed within 3 months.	Strong consensus	Strong consensus
31	When adding CBD to a regimen containing STP, VPA, and CLB, if a partial seizure improvement and/or SE reduction was observed following STP addition and prior to CBD initiation, maintaining STP should be considered.	Good consensus	Strong consensus
32	When adding CBD to any base regimen of ASM, including VPA, transaminase levels should be evaluated at baseline and monitored during titration because of the potential for transaminitis in patients on combined VPA and CBD.	Strong consensus	Strong consensus
33	When introducing CBD to a regimen containing STP, VPA, and CLB, the most common adverse effects include sedation, somnolence, fatigue, ataxia, diarrhea, nausea, and anorexia. These are often transient, but if present, a lower target CBD dose and slower CBD titration could be considered.	Good consensus	Strong consensus
**Patient follow‐up during treatment initiation**			
34	After adding a new treatment, an initial follow‐up via a nurse and/or physician contact, telephone, or secure email within 1–2 weeks to assess new treatment's safety and the family's confidence in the new ASM regimen may be considered.	Strong consensus	Strong consensus
35	After adding a new treatment, a follow‐up visit should be scheduled at 1–3 months post‐initiation to assess safety and efficacy.	Strong consensus	Strong consensus
36	When STP is added to CLB and VPA, blood counts and liver function should be assessed prior to starting treatment with STP and should be checked every 6 months unless otherwise clinically indicated.	Good consensus	Strong consensus
37	When FFA is added to any regimen, to detect aortic or mitral valvular heart disease and pulmonary arterial hypertension, an echocardiogram should be performed at baseline and every 6 months after initiation for the first two years and then eventually annually according to local regulations. Also, a final echocardiogram should be conducted 3–6 months after the last dose of treatment with FFA.	Strong consensus	Strong consensus
38	When CBD is added to a regimen containing VPA, liver transaminases (AST, ALT) should be assessed at baseline and at 6 weeks post‐initiation due to the potential interaction between CBD and VPA, which may lead to elevated transaminase levels. A follow‐up assessment of AST and ALT levels should also be conducted at 3 months.	Strong consensus	Strong consensus

Abbreviations: ASM, anti‐seizure medication, CBD, cannabidiol, CLB, clobazam, FFA, fenfluramine, LEV, levetiracetam, STP, stiripentol, TPM, topiramate.

Overall, 34 of the 38 statements (89%) reached strong or good consensus among both the nominal group experts and the international voting panel; one did not reach consensus in either group; and 3 reached consensus only among the nominal group experts (Table 1).

### 3.1 General principles (7 statements)

Six statements achieved consensus in both groups. Experts agreed that all individuals with DS should have rescue medications and a defined seizure plan (statement #1), that treatment optimization ‐ balancing efficacy and tolerability ‐ should precede any regimen change (#2), and that sufficient time should be allowed to assess the effectiveness of newly introduced therapies (#3). Routine ASM plasma level monitoring when introducing STP or CBD was considered unnecessary, as clinical assessment is generally sufficient to monitor potential adverse effects and efficacy (#5). Experts also agreed on a tailored management of comorbidities (#6) and on avoiding sodium channel blockers in a confirmed DS diagnosis (#4).

One statement was discordant between the groups (strong consensus among the nominal group expert vs no consensus among the international DS specialists): withdrawal of a previously ineffective ASM over 4–8 weeks after STP initiation (#7).

### 3.2 Guiding physician to empower patients (4 statements)

Strong consensus for all statements was reached in both groups.

Experts agreed that physicians should actively involve caregivers in treatment decisions (#8), set clear expectations on efficacy and adverse effects when initiating a new treatment (#10), provide a structured management plan to caregivers (#11), and discuss possible clinical trial participation when appropriate (#9).

### 3.3 STP as an add‐on to specific and non‐specific Dravet ASMs (10 statements)

Nine statements reached a strong consensus among both groups: experts agreed that STP should be introduced to a regimen in combination with VPA and/or CLB (#13). They recommended initiating STP at 15–20 mg/kg/day and gradually titrating over 2–4 weeks to a target dose adjusted for age, weight, seizure frequency, efficacy, and tolerability (#14). Children under 5 years of age may require higher per kilogram doses (up to 50 mg/kg/day) than adolescents (around 30 mg/kg/day) (#15). Early dose reduction of VPA and CLB at STP initiation was advised to minimize adverse effects (#16).

Specifically, regarding STP interaction with other ASMs when introduced:

1. For CLB, experts recommended not exceeding 0.5 mg/kg/day and reducing its dose by 25% if adverse effects occur (#17).
2. For VPA, the dose should not be higher than 30 mg/kg/day, with a 25% dose reduction if side effects are observed (#18).
3. For FFA, STP introduction should prompt FFA dose adjustment to a maximum of 0.4 mg/kg/day, not exceeding 17 mg/day (#19).
4. For CBD, no dose change of either drug was considered necessary. Experts also agreed that, as the addition of STP is not expected to further influence the pharmacokinetics of co‐medications such as CLB, no additional adjustment was recommended, but that careful clinical monitoring remains essential (#20).
5. When either TPM or LEV is co‐administered, no dose adjustment of either agent was considered necessary (#21).

One statement showed discordance between the two groups: the nominal group experts, but not the international DS specialists, strongly agreed that when STP is added to a regimen including VPA and/or CLB, a meaningful reduction in seizure frequency and duration is generally expected within 4 to 6 weeks after reaching the target dose (#12).

### 3.4 FFA in a regimen containing STP (5 statements)

All statements reached consensus among both groups. Experts agreed that introducing FFA to a regimen containing STP is generally associated with a significant reduction in seizure frequency within 4–6 weeks after achieving the target dose (#22). There was also a strong consensus that echocardiographic evaluation is mandatory before initiating FFA to exclude valvular heart disease and pulmonary arterial hypertension (#24). Regarding FFA initiation in a regimen containing STP, experts recommended starting FFA at 0.1–0.2 mg/kg/day, and titrating by 0.1 mg/kg/day every 1–2 weeks based on response and tolerability, up to a maximum of 0.4 mg/kg/day, not exceeding 17 mg/day (#23). Two additional statements reached consensus:

1. STP should generally be maintained if seizure improvement and/or reduction of status epilepticus was observed prior to FFA addition (#25).
2. Adverse effects can occur but are often transient; lower titration and a lower FFA target dose may enhance tolerability (#26).

### 3.5 CBD in a regimen containing STP (7 statements)

Five statements reached consensus in both groups; one did not achieve consensus in either group, and one reached consensus among the nominal group but not among the international DS specialists.

Regarding the initiation of CBD to a regimen containing STP, there was strong consensus that treatment should start at 2.5 mg/kg/day, followed by weekly increases of 2 mg/kg/day to reach an initial target dose of 10–12 mg/kg/day over 3–4 weeks. Experts agreed that if seizures persist and the drug is well tolerated, further increases of 2.5 mg/kg/day every 1–2 weeks can be considered, up to a maximum dose of 20 mg/kg/day (#28). Evaluating the clinical benefit of CBD within 3 months after initiation was also consensual (#30).

Regarding the management of co‐medications and adverse effects:

1. Interestingly, the two groups disagreed on whether an additional dose adjustment of CLB is required when CBD is added to a regimen already containing STP and CLB, considering the pharmacokinetic interaction STP already has with CLB (#29): the nominal group experts reached consensus that no further adjustment was needed, while the international group disagreed, suggesting that the addition of CBD could require a dose change.
2. However, there was strong consensus that in patients receiving both VPA and CBD, liver transaminases should be measured at baseline and monitored during titration due to the risk of elevation (#32).
3. Experts agreed that common adverse effects observed when introducing CBD are often transient. If these occur, experts recommend a slower titration and lower target dose of CBD to improve tolerability (#33).
4. They also agreed that if STP had led to partial improvement before CBD was introduced, it should generally be maintained in the regimen (#31).

In contrast, neither group reached consensus on whether adding CBD to a regimen that includes STP results in a moderate reduction in overall seizure frequency (#27).

### 3.6 Patient follow‐up during treatment initiation (5 statements)

All statements reached consensus among both groups. Experts recommended an early follow‐up within 1–2 weeks after treatment initiation to assess initial safety and support families adapting to a new regimen (#34). A subsequent visit between 1 and 3 months after treatment initiation should evaluate both efficacy and tolerability (#35). Treatment‐specific monitoring was also emphasized: for patients receiving FFA, baseline echocardiography and periodic cardiac evaluations every 6 months during the first 2 years, then annually, and once after discontinuation were advised (#37). For CBD combined with VPA, liver function tests should be performed at baseline, 6 weeks, and 3 months to detect potential transaminase elevations (#38). When STP is used with CLB and VPA, baseline blood count and liver function tests are recommended, followed by reassessment every 6 months unless otherwise indicated (#36).

## 4 DISCUSSION

The drug‐resistant nature of DS has led to an expanding range of treatment options and the frequent use of polytherapy. Combined with the need for early intervention, these factors contribute to a highly complex therapeutic landscape for clinicians.^11^, ^20^ This consensus initiative aimed to support clinicians in optimizing the management of multi‐drug therapy for DS by integrating evidence‐based data with expert clinical insights.

To this end, we used the Nominal Group Technique, a structured and recognized consensus methodology,^25^, ^26^ followed by a broader vote among DS specialists. Among the 38 statements formulated, 34 reached strong or good consensus in both groups, 1 failed to reach consensus in both groups, and 3 did not reach consensus within the international DS specialists but did within the expert group.

One key aspect of DS management is the need for strong communication with parents and caregivers. Given the severity and complexity of the disease and its broad impact on the entire family, adopting an individualized approach to better understand caregiver expectations is essential. Despite its challenges, this approach enables clinicians to provide families with clear, comprehensive, and accessible informations^11^, ^31^ In this regard, all statements from sections 2 (“Guiding physicians to empower parents or caregivers”) and 6 (“Patient follow‐up during treatment initiation”) reached strong consensus across both voting groups, underscoring the importance of actively involving families in treatment decisions and follow‐up. These findings are consistent with the Delphi study by Brunklaus et al., which highlighted the critical need for structured communication at the time of diagnosis.^22^ Our results further confirm the central role of caregiver empowerment in the effective management of DS.

Another critical aspect of DS management lies in balancing seizure control with treatment safety and quality of life.^32^ In our study, the statement recommending tapering off an ineffective ASM following the introduction of STP (statement #7) reached strong consensus among the nominal group experts but not among the international DS specialists—although the median score (7/9) suggests near‐consensus. This aligns with findings from the Delphi consensus by Wirrell et al.,^6^ where strong agreement was reached on reducing polypharmacy by discontinuing less effective ASMs when newer agents improve seizure control, implying that treatment optimization should prioritize the retention of ASMs that demonstrate clear clinical benefit while considering the withdrawal of those with limited or no efficacy.^12^ In our work, the lack of consensus observed within the international panel may reflect the practical difficulty of identifying, within complex polytherapy regimens, which individual ASMs are truly ineffective. Prospective studies specifically designed to evaluate ASM withdrawal will be needed to provide higher‐quality evidence and clarify the therapeutic value of each drug within defined treatment regimens.

Interestingly, consistent with the recommendation to taper off ineffective ASMs and retain effective ones, both panels reached consensus on maintaining STP in the regimen when introducing FFA or CBD (statement #25 and #31), provided STP had demonstrated efficacy on seizures and status epilepticus. STP's efficacy on these aspects is well documented in the literature,^13^, ^14^, ^17^, ^33^, ^34^ which underscores the importance of retaining proven therapies while discontinuing those with limited efficacy.

Managing DS is particularly complex due to the diversity and heterogeneity of available treatment options, which require clinicians to consider multiple parameters when introducing a new ASM, such as efficacy, potential drug–drug interactions, pharmacokinetic profiles, pediatric‐friendly formulations, and the management of adverse effects.^21^ In our study, consensus was reached across both panels on key aspects related to the introduction of a new ASM, including initiation parameters (starting dose, titration, target dose), adjustments to existing treatments (e.g., reducing FFA or CBD when adding STP), and the management of adverse events. These key parameters addressed in our consensus are consistent with previously published recommendations^11^, ^14^, ^35^, ^36^ and the official product characteristics of the drugs concerned.^37^, ^38^, ^39^, ^40^ Overall, this agreement reflects a high level of expert convergence on these complex but essential aspects of care, supporting the development of practical, experience‐based recommendations for the optimization of polytherapy management in DS.

In this study, several points of disagreement emerged between the nominal group experts and the international DS specialists. The first concerned drug–drug interactions, specifically the pharmacokinetic interplay between STP, CLB, and CBD (statement #29). The international DS specialists did not reach consensus on whether CLB dosage should be further reduced when CBD is added to a patient already receiving STP and CLB (although the median voting score of 7 indicates a near consensus). A previously published study by Devinsky et al. showed that STP maximally inhibits CYP450 enzymes involved in CLB metabolism and that adding CBD did not further alter CLB or norclobazam (the active metabolite of CLB) levels in that small subgroup (*n* = 4). While these findings support maintaining the CLB dose in this context—combined with close clinical surveillance and, when appropriate, monitoring of concomitant ASM plasma concentrations—the absence of additional pharmacokinetic interactions requires confirmation in a larger cohort. This reflects the complexity of drug–drug interactions in DS management and the importance of considering available pharmacological data when adjusting polytherapy regimens.

Other disagreements between the two groups related to statements on the expected efficacy of DS‐specific ASMs (e.g. STP, FFA, and CBD). In particular, international DS specialists did not reach consensus on the expected efficacy of STP in controlling seizures and status epilepticus when added to an existing regimen (statement #12), despite robust supporting evidence.^13^, ^14^, ^17^, ^33^, ^34^ Notably, the median score (7.5/9) indicated near‐consensus. Since panelists could not provide written comments, the reasons for disagreement remain unclear. One possible explanation lies in the complexity of the statement formulation, which combined multiple sub‐elements (efficacy on seizures, efficacy on SE, and the expected timeframe to respond within 4–6 weeks). Splitting this statement into distinct concepts may have facilitated consensus. This consideration also applies to other statements conveying more than one distinct message (e.g., statements #5, #17, #18, etc.). Another possible explanation for the lack of consensus within the international panel may be the variability in treatment response among patients with DS, which can lead clinicians to be more cautious when considering the expected efficacy of a given therapy.

Similarly, the statement regarding the expected efficacy of CBD was the only one that did not reach consensus in either group (statement #27). Here again, as DS specialists were not able to comment on their vote, the interpretation of this result is limited. The use of the term “moderate” efficacy rather than “significant” efficacy may have influenced responses. Here also, it is worth noting that the statement approached near‐consensus, with a median score of 7/9 and 63.5% expert agreement. This result may reflect clinical trial data showing lower seizure reduction with CBD compared to the other DS‐specific ASMs and its frequent positioning as a third‐line option.^11^, ^16^, ^41^, ^42^ Interestingly, in the international consensus conducted by Wirrell et al.,^6^ only 31% of physicians considered CBD a first‐ or second‐line option, compared to 86% of caregivers, highlighting the ongoing challenge for clinicians in managing caregivers' beliefs and understandings and the need to strengthen communication to better address family expectations and treatment demands.

As with all consensus methodologies, this study has limitations. The NGT voting panel included a limited number of experts (4), which may restrict the representativeness of its vote.^26^ To address this, we expanded the voting process to a broader group of clinicians. However, the MasterClass voting context also introduced limitations: participants voted without open discussion or the opportunity to provide a rationale, which complicates the interpretation of the voting outcomes, especially for more complex or ambiguously worded statements. Additionally, geographical variability in drug availability may have influenced voting behavior in the international voting panel.

## 5 CONCLUSION

The inherent variability of DS needs individualized treatment approaches, which remain the standard of care for this DEE, with therapeutic approaches that should be adapted to treatment response, tolerability, and clinical judgment. To our knowledge, this consensus initiative is the first to integrate both evidence‐based data and expert clinical insights to support those individualized approaches, by establishing common principles to guide clinicians in the management of multidrug regimens, particularly those less familiar with STP use or DS polytherapy. Overall, 34 out of 38 statements (89%) reached consensus, notably on caregiver empowerment, initiation and titration parameters for key ASMs, drug–drug interaction management, and treatment monitoring. These areas of agreement form a solid foundation of practical guidance for clinical decision‐making and to help clinicians communicate more effectively with families about the goals and implications of combination therapy in DS.

Our work also highlights the complexity of pharmacological interactions in ASM polytherapy and emphasizes the need to stay current with the literature to optimize patient care. As most voters were from Europe (90%), expanding the consensus process to include larger and more diverse expert panels, particularly from North America and other underrepresented regions, would help reinforce and refine these recommendations, contributing to the establishment of consistent international guidance for STP‐based treatment strategies in DS.

## AUTHOR CONTRIBUTIONS

All authors were part of the Steering Committee and equally contributed to this study: defined the final scope of the study, reviewed literature, formulated (or reformulated), and voted on statements. They all critically reviewed the manuscript for intellectual content, integrated revisions, and agreed to the publication of the manuscript.

## CONFLICT OF INTEREST STATEMENT

J. Helen Cross has acted as an investigator for studies with Jazz/GW Pharmaceuticals, Stoke Therapeutics, UCB/Zogenix, Ultragenyx, Encoded Epigenyx, and Lundbeck; has been a speaker and has served on advisory boards for Biocodex, Jazz Pharmaceuticals, Nutricia, Stoke Therapeutics, and UCB (all remuneration has been paid to her department); holds an endowed chair at the University College of London Great Ormond Street Institute of Child Health; has received grants from the National Institute for Health and Care Research (NIHR), the Engineering and Physical Sciences Research Council (EPSRC), the Great Ormond Street Hospital for Children (GOSH) Charity, LifeArc, and Epilepsy Institute UK; and her research is supported by the NIHR Great Ormond Street Hospital Biomedical Research Centre. Rima Nabbout has received compensation for consulting work and/or attending Scientific Advisory Boards from GW Pharma, LivaNova, Lundbeck, Marinus, Stoke, Supernus, Advicenne, Takeda, UCB Inc., Servier, Eisai, Ionis, Zogenix, and Neuraxpharm. She has research grants from the European FP7 program and the European joint program on rare diseases. Elaine Wirrell has received consulting fees from Biocodex and Jazz Pharma. She serves on the DSMB for Neurocrine, GRIN pharma, Acadia, and Encoded. Barry Gidal has received honoraria from AUCTA, Neurelis, UCB, SK Life Science, and Jazz. Joseph Sullivan has received research funding from Zogenix, UCB, Stoke Therapeutics, Encoded Therapeutics, Xenon Pharmaceuticals, Neurocrine Biosciences, and Takeda. He has served as a consultant for the Epilepsy Study Consortium, Jazz Pharmaceuticals, UCB Pharma, Marinus Pharmaceuticals, BrightMinds, Longboard, and Ceribell. He serves on the DSMB for Neuropace. He also holds restricted stock units from Zynerba. The authors confirm that they have read the Journal's position on issues involved in ethical publication and affirm that this report is consistent with those guidelines.

## Supporting information


Data S1

## Data Availability

The data that support the findings of this study are available from the corresponding author upon reasonable request.
