# Supplementary material for: Practical consensus recommendations for polytherapy involving stiripentol in Dravet syndrome: A nominal group approach
Source: Epilepsia Open. 2026 Mar 18;11(3):833–46. doi: 10.1002/epi4.70245 (PMC13238850; doi:10.1002/epi4.70245)
Supplement: Supplementary file 1 — Data S1 [file EPI4-11-833-s001.docx]

**Supplementary Table S1:** Voting results from the Nominal Group experts

|  | **Statements** | **Scores 1-2-3 (n)** | **Scores 4-5-6 (n)** | **Scores 7-8-9 (n)** | **Median** | **Results** |
| --- | --- | --- | --- | --- | --- | --- |
| **General principles** | | | | | | |
| 1 | Given propensity for prolonged seizure in Dravet Syndrome, every patient should be prescribed a rescue medication, and a clear seizure action plan should be provided. | 0%  (0) | 0%  (0) | 100% (4) | 9 | **Strong consensus** |
| 2 | Before considering a switch or introducing a new medication, priority should be given to optimizing the dosage of an existing treatment taking into consideration efficacy and tolerability. | 0%  (0) | 0%  (0) | 100% (4) | 8.5 | **Strong consensus** |
| 3 | When a new treatment is introduced and adjustments are made to the ASM regimen, a change in seizure frequency  and/or severity may occur. A sufficient time should be given to evaluate effectiveness (efficacy and tolerability)  of the new treatment. | 0%  (0) | 0%  (0) | 100% (4) | 8.5 | **Strong consensus** |
| 4 | Sodium Channel Blocking ASM should be avoided following a diagnosis of Dravet Syndrome. | 0%  (0) | 0%  (0) | 100% (4) | 9 | **Strong consensus** |
| 5 | When adding STP or CBD to any base regimen, potential adverse effects can usually be assessed clinically, and plasma levels are not mandatory to optimize treatment effectiveness (efficacy and tolerability). | 0%  (0) | 0%  (0) | 100% (4) | 8.5 | **Strong consensus** |
| 6 | ASM changes may partly impact comorbidities, however the underlying causes of comorbidities are multifactorial. Comorbidities require ongoing evaluation and targeted intervention. | 0%  (0) | 0%  (0) | 100% (4) | 9 | **Strong consensus** |
| 7 | Following introduction of STP, if an existing ASM did not afford significant seizure improvement, then tapering it off over 4–8 weeks should be considered. | 0% (0) | 25% (1) | 75% (3) | 8 | **Strong consensus** |
| **Guiding physician to empower parents or caregiver** | | | | | | |
| 8 | Physicians should actively involve parents or caregivers in treatment decisions and discuss of the advantages  and disadvantages of the various available options, including ASM, dietary therapies and neuromodulation. | 0%  (0) | 0%  (0) | 100% (4) | 8.5 | **Strong consensus** |
| 9 | Physicians should discuss with parents or caregivers about referral and potential inclusion in clinical trials. | 0%  (0) | 0%  (0) | 100% (4) | 9 | **Strong consensus** |
| 10 | When initiating a new treatment, physician should give parents or caregiver clear expectations regarding treatment efficacy and possible adverse effects. | 0%  (0) | 0%  (0) | 100% (4) | 9 | **Strong consensus** |
| 11 | Physician should provide parents or caregivers with a clear plan including written information regarding medication titration, tapering, as well as guidance on monitoring seizures and adverse effects, and when and how to seek medical advice. | 0%  (0) | 0%  (0) | 100% (4) | 9 | **Strong consensus** |
| **STP as an add-on to other ASMs** | | | | | | |
| 12 | When adding STP to any base regimen of ASM including VPA and/or CLB, a significant reduction in seizure frequency and duration, as well as a significant decrease or a cessation in episodes of prolonged convulsive seizures and status epilepticus is generally anticipated within 4 to 6 weeks after reaching the target dose. | 0%  (0) | 0%  (0) | 100% (4) | 8 | **Strong consensus** |
| 13 | STP should be introduced to a regimen containing VPA and/or CLB. | 0%  (0) | 0%  (0) | 100% (4) | 8 | **Strong consensus** |
| 14 | When adding STP to any base regimen of ASM, initiation at 15-20 mg/kg/day is recommended, with gradual increase over 2–4 weeks to the initial target dose, adjusted based on seizure frequency, efficacy, and tolerability. | 0%  (0) | 0%  (0) | 100% (4) | 8.5 | **Strong consensus** |
| 15 | When adding STP to any base regimen of ASM, the weight and age of the patient must be considered: children younger than 5 years old usually require higher targeted dose (50 mg/kg/day), whereas adolescents require lower targeted dose (30 mg/kg/day). | 0%  (0) | 0%  (0) | 100% (4) | 9 | **Strong consensus** |
| 16 | When adding STP to a base regimen containing VPA and CLB, early reduction of co-medications at the initiation of STP should be considered to help minimize the occurrence of adverse effects, as they are most likely due to higher levels of those 2 co-medications. | 0%  (0) | 0%  (0) | 100% (4) | 8.5 | **Strong  consensus** |
| 17 | Considering drug interactions with CLB, when STP is introduced:   - If the regimen already contains CLB, adjusting CLB to a maximum 0.5 mg/kg/day should be considered. - If adverse effects such as sedation, somnolence, ataxia are observed, tolerability can be improved  by decreasing CLB by 25%. | 0%  (0) | 0%  (0) | 100% (4) | 8.5 | **Strong  consensus** |
| 18 | Considering drug interactions with VPA, when STP is introduced:   - If the regimen already contains VPA, adjusting VPA to a maximum of 30 mg/kg/day should be considered. - If adverse effects such as sedation, decrease appetite, weight loss or thrombocytopenia are observed, tolerability can be improved by decreasing VPA by 25%. | 0%  (0) | 0%  (0) | 100% (4) | 8.5 | **Strong  consensus** |
| 19 | Considering drug interactions with FFA, when STP is introduced: FFA must be adjusted to a maximum of 0.4 mg/kg/day, not exceeding 17 mg/day. | 25% (1) | 0% (0) | 75% (3) | 9 | **Strong  consensus** |
| 20 | Considering drug interactions with CBD, when STP is introduced:   - No specific dose adjustment of CBD nor STP is required. - The addition of STP is not expected to further induce pharmacokinetic interactions with concomitant co-medications, such as CLB. Therefore, no further adjustment of CLB is required; however, careful clinical monitoring is recommended | 0%  (0) | 0%  (0) | 100% (4) | 8.5 | **Strong  consensus** |
| 21 | Considering STP drug interactions with TPM or LEV, no specific dose adjustment is required. | 0%  (0) | 0%  (0) | 100% (4) | 9 | **Strong  consensus** |
| **FFA in a regimen containing STP** | | | | | | |
| 22 | When adding FFA to any base regimen of ASM including STP, a significant reduction of seizures frequency is generally anticipated within 4 to 6 weeks after reaching the target dose. | 0%  (0) | 0%  (0) | 100% (4) | 8 | **Strong  consensus** |
| 23 | When adding FFA to any base regimen of ASM including STP, an initial dose of 0.1–0.2 mg/kg/day is recommended, with gradual titration by 0.1 mg/kg/day every 1–2 weeks based on treatment response and tolerability, aiming for a maximum dose of 0.4 mg/kg/day, not exceeding 17 mg/day. | 0%  (0) | 0%  (0) | 100% (4) | 9 | **Strong  consensus** |
| 24 | Before introducing FFA to any base regimen, an echocardiography must be performed to confirm the absence of aortic or mitral valvular heart disease and pulmonary arterial hypertension. | 0%  (0) | 0%  (0) | 100% (4) | 9 | **Strong  consensus** |
| 25 | When adding FFA to a regimen containing STP, VPA, and CLB, if a partial seizure improvement and/or SE reduction was observed following STP addition and prior to FFA initiation, maintaining STP should be considered. | 0%  (0) | 0%  (0) | 100% (4) | 7 | Good consensus |
| 26 | When adding FFA to a base regimen containing STP, VPA, and CLB, common adverse effects include decreased appetite, fatigue and somnolence. These are often transient, but if present, lower FFA target dose and slower FFA titration could be considered. | 0%  (0) | 25%  (1) | 75% (3) | 7.5 | Good consensus |
| **CBD in a regimen containing STP** | | | | | | |
| 27 | When adding CBD to any base regimen of ASM including STP, a moderate reduction in overall seizures frequency is generally anticipated. | 0%  (0) | 75% (3) | 25%  (1) | 6 | **No Consensus** |
| 28 | When adding CBD to any base regimen of ASM including STP, initiation at 2.5 mg/kg/day is recommended, with 2 mg/kg/day weekly increases to an initial target dose of 10-12 mg/kg/day over 3–4 weeks. Treatment response should then be assessed, with further increases of 2.5 mg/kg/day every 1–2 weeks if seizures persist, up to a maximum dose of 20 mg/kg/day if well tolerated. | 0% (0) | 25% (1) | 75% (3) | 8.5 | **Strong consensus** |
| 29 | Since STP already has a pharmacokinetic interaction with CLB, no further dose reduction of CLB is necessary when adding CBD to a patient already receiving STP and CLB. | 0%  (0) | 0%  (0) | 100% (4) | 9 | **Strong  consensus** |
| 30 | When adding CBD to any base regimen of ASM including STP, the benefit of CBD should be assessed within 3 months. | 0%  (0) | 0%  (0) | 100% (4) | 8 | **Strong  consensus** |
| 31 | When adding CBD to a regimen containing STP, VPA, and CLB, if a partial seizure improvement and/or SE reduction was observed following STP addition and prior to CBD initiation, maintaining STP should be considered. | 0%  (0) | 0%  (0) | 100% (4) | 7 | Good consensus |
| 32 | When adding CBD to any base regimen of ASM including VPA, transaminase levels should be evaluated at baseline  and monitored during titration because of the potential for transaminitis in patients on combined VPA and CBD. | 0%  (0) | 0%  (0) | 100% (4) | 8.5 | **Strong  consensus** |
| 33 | When introducing CBD to a regimen containing STP, VPA, and CLB, most common adverse effects include sedation, somnolence, fatigue, ataxia, diarrhea, nausea, and anorexia. These are often transient, but if present, lower target CBD dose and slower CBD titration could be considered. | 0% (0) | 25% (1) | 75% (3) | 7.5 | Good consensus |
| **Patient follow-up during treatment initiation** | | | | | | |
| 34 | After adding a new treatment, an initial follow-up via a nurse and/or physician contact, telephone, or secure email within 1–2 weeks to assess new treatment’s safety and the family's confidence in the new ASM regimen may be considered. | 0%  (0) | 0%  (0) | 100% (4) | 8 | **Strong  consensus** |
| 35 | After adding a new treatment, a follow-up visit should be scheduled at 1–3 months post-initiation to assess safety and efficacy. | 0%  (0) | 0%  (0) | 100% (4) | 9 | **Strong  consensus** |
| 36 | When STP is added to CLB and VPA, blood counts and liver function should be assessed prior to starting treatment with STP and should be checked every 6 months unless otherwise clinically indicated. | 0% (0) | 25% (1) | 75% (3) | 7 | Good consensus |
| 37 | When FFA is added to any regimen, and to detect aortic or mitral valvular heart disease and pulmonary arterial hypertension, an echocardiogram should be performed at baseline and every 6 months after initiation for the first two years, and then eventually annually according to local regulation. Also, a final echocardiogram should be conducted 3-6 months after the last dose of treatment with FFA. | 0%  (0) | 0%  (0) | 100% (4) | 8.5 | **Strong  consensus** |
| 38 | When CBD is added to a regimen containing VPA, liver transaminases (AST, ALT) should be assessed at baseline  and at 6 weeks post-initiation due to the potential interaction between CBD and VPA, which may lead to elevated transaminase levels. A follow-up assessment of AST and ALT levels should also be conducted at 3 months. | 0% (0) | 25% (1) | 75% (3) | 8 | **Strong consensus** |

%1-2-3 represents the percentage of votes ≤3; n represents absolute number

%4-5-6 represents the percentage of votes ≥4 and ≤6; n represents absolute number

%7-8-9 represents the percentage of votes ≥7; n represents absolute number

**Supplementary Table S2:** Results of the reinforcement vote by the international expert panel

*As responses to the questions were optional, the total number of respondents varies across items.*

|  | **Statements** | **Scores 1-2-3 (n)** | **Scores 4-5-6 (n)** | **Scores 7-8-9 (n)** | **Median** | **Results** |
| --- | --- | --- | --- | --- | --- | --- |
| **General principles** | | | | | | |
| 1 | Given propensity for prolonged seizure in Dravet Syndrome, every patient should be prescribed a rescue medication, and a clear seizure action plan should be provided. | 0%  (0) | 0%  (0) | 100% (53) | 9 | **Strong consensus** |
| 2 | Before considering a switch or introducing a new medication, priority should be given to optimizing the dosage of an existing treatment taking into consideration efficacy and tolerability. | 0%  (0) | 9.1%  (5) | 90.9% (50) | 9 | **Strong consensus** |
| 3 | When a new treatment is introduced and adjustments are made to the ASM regimen, a change in seizure frequency  and/or severity may occur. A sufficient time should be given to evaluate effectiveness (efficacy and tolerability)  of the new treatment. | 0%  (0) | 1.9%  (1) | 98.1% (53) | 8.5 | **Strong consensus** |
| 4 | Sodium Channel Blocking ASM should be avoided following a diagnosis of Dravet Syndrome. | 0%  (0) | 1.8%  (1) | 98.2% (55) | 9 | **Strong consensus** |
| 5 | When adding STP or CBD to any base regimen, potential adverse effects can usually be assessed clinically,and plasma levels are not mandatory to optimize treatment effectiveness (efficacy and tolerability). | 13%  (7) | 20.3%  (11) | 66.7% (36) | 8 | Good consensus |
| 6 | ASM changes may partly impact comorbidities, however the underlying causes of comorbidities are multifactorial. Comorbidities require ongoing evaluation and targeted intervention. | 0%  (0) | 1.9%  (1) | 98.1% (51) | 9 | **Strong consensus** |
| 7 | Following introduction of STP, if an existing ASM did not afford significant seizure improvement, then tapering it off over 4–8 weeks should be considered. | 9.4% (5) | 26.4% (14) | 64.2% (34) | 7 | **No Consensus** |
| **Guiding physician to empower parents or caregiver** | | | | | | |
| 8 | Physicians should actively involve parents or caregivers in treatment decisions and discuss of the advantages  and disadvantages of the various available options, including ASM, dietary therapies and neuromodulation. | 0%  (0) | 1.9%  (1) | 98.1% (52) | 9 | **Strong consensus** |
| 9 | Physicians should discuss with parents or caregivers about referral and potential inclusion in clinical trials. | 0%  (0) | 0%  (0) | 100% (54) | 9 | **Strong consensus** |
| 10 | When initiating a new treatment, physician should give parents or caregiver clear expectations regarding treatment efficacy and possible adverse effects. | 0%  (0) | 1.8%  (1) | 98.2% (54) | 9 | **Strong consensus** |
| 11 | Physician should provide parents or caregivers with a clear plan including written information regarding medication titration, tapering, as well as guidance on monitoring seizures and adverse effects, and when and how to seek medical advice. | 0%  (0) | 3.8%  (2) | 96.2% (51) | 9 | **Strong consensus** |
| **STP as an add-on to other ASMs** | | | | | | |
| 12 | When adding STP to any base regimen of ASM including VPA and/or CLB, a significant reduction in seizure frequency and duration, as well as a significant decrease or a cessation in episodes of prolonged convulsive seizures and status epilepticus is generally anticipated within 4 to 6 weeks after reaching the target dose. | 0%  (0) | 28.6%  (16) | 71.4% (40) | 7.5 | **No Consensus** |
| 13 | STP should be introduced to a regimen containing VPA and/or CLB. | 0%  (0) | 11.1%  (6) | 88.9% (48) | 8 | **Strong consensus** |
| 14 | When adding STP to any base regimen of ASM, initiation at 15-20 mg/kg/day is recommended, with gradual increase over 2–4 weeks to the initial target dose, adjusted based on seizure frequency, efficacy, and tolerability. | 3.6%  (2) | 12.7%  (7) | 83.7% (46) | 8 | **Strong consensus** |
| 15 | When adding STP to any base regimen of ASM, the weight and age of the patient must be considered: children younger than 5 years old usually require higher targeted dose (50 mg/kg/day), whereas adolescents require lower targeted dose (30 mg/kg/day). | 0%  (0) | 9.1%  (5) | 90.9% (50) | 8 | **Strong consensus** |
| 16 | When adding STP to a base regimen containing VPA and CLB, early reduction of co-medications at the initiation of STP should be considered to help minimize the occurrence of adverse effects, as they are most likely due to higher levels of those 2 co-medications. | 1.9%  (1) | 1.9%  (1) | 96.2% (52) | 8 | **Strong  consensus** |
| 17 | Considering drug interactions with CLB, when STP is introduced:   - If the regimen already contains CLB, adjusting CLB to a maximum 0.5 mg/kg/day should be considered. - If adverse effects such as sedation, somnolence, ataxia are observed, tolerability can be improved  by decreasing CLB by 25%. | 1.8%  (1) | 9.1%  (5) | 89.1% (49) | 8 | **Strong  consensus** |
| 18 | Considering drug interactions with VPA, when STP is introduced:   - If the regimen already contains VPA, adjusting VPA to a maximum of 30 mg/kg/day should be considered. - If adverse effects such as sedation, decrease appetite, weight loss or thrombocytopenia are observed, tolerability can be improved by decreasing VPA by 25%. | 7.4%  (4) | 16.7%  (9) | 75.9% (41) | 8 | **Strong  consensus** |
| 19 | Considering drug interactions with FFA, when STP is introduced: FFA must be adjusted to a maximum of 0.4 mg/kg/day, not exceeding 17 mg/day. | 1.8% (1) | 1.8% (1) | 96.4% (54) | 9 | **Strong  consensus** |
| 20 | Considering drug interactions with CBD, when STP is introduced:   - No specific dose adjustment of CBD nor STP is required. - The addition of STP is not expected to further induce pharmacokinetic interactions with concomitant co-medications, such as CLB. Therefore, no further adjustment of CLB is required; however, careful clinical monitoring is recommended | 14.3%  (8) | 7.1%  (4) | 78.6% (44) | 8 | **Strong  consensus** |
| 21 | Considering STP drug interactions with TPM or LEV, no specific dose adjustment is required. | 0%  (0) | 12.7%  (7) | 87.3% (48) | 8 | **Strong  consensus** |
| **FFA in a regimen containing STP** | | | | | | |
| 22 | When adding FFA to any base regimen of ASM including STP, a significant reduction of seizures frequency is generally anticipated within 4 to 6 weeks after reaching the target dose. | 1.9%  (1) | 14.8%  (8) | 83.3% (45) | 8 | **Strong  consensus** |
| 23 | When adding FFA to any base regimen of ASM including STP, an initial dose of 0.1–0.2 mg/kg/day is recommended, with gradual titration by 0.1 mg/kg/day every 1–2 weeks based on treatment response and tolerability, aiming for a maximum dose of 0.4 mg/kg/day, not exceeding 17 mg/day. | 1.8%  (1) | 5.5%  (3) | 92.7% (51) | 9 | **Strong  consensus** |
| 24 | Before introducing FFA to any base regimen, an echocardiography must be performed to confirm the absence of aortic or mitral valvular heart disease and pulmonary arterial hypertension. | 0%  (0) | 0%  (0) | 100% (50) | 9 | **Strong  consensus** |
| 25 | When adding FFA to a regimen containing STP, VPA, and CLB, if a partial seizure improvement and/or SE reduction was observed following STP addition and prior to FFA initiation, maintaining STP should be considered. | 0%  (0) | 7.4%  (4) | 92.6% (50) | 8 | **Strong  consensus** |
| 26 | When adding FFA to a base regimen containing STP, VPA, and CLB, common adverse effects include decreased appetite, fatigue and somnolence. These are often transient, but if present, lower FFA target dose and slower FFA titration could be considered. | 0%  (0) | 25%  (1) | 75% (3) | 7.5 | Good consensus |
| **CBD in a regimen containing STP** | | | | | | |
| 27 | When adding CBD to any base regimen of ASM including STP, a moderate reduction in overall seizures frequency is generally anticipated. | 5.5%  (3) | 30.9% (17) | 63.6%  (35) | 7 | **No Consensus** |
| 28 | When adding CBD to any base regimen of ASM including STP, initiation at 2.5 mg/kg/day is recommended, with 2 mg/kg/day weekly increases to an initial target dose of 10-12 mg/kg/day over 3–4 weeks. Treatment response should then be assessed, with further increases of 2.5 mg/kg/day every 1–2 weeks if seizures persist, up to a maximum dose of 20 mg/kg/day if well tolerated. | 10.7% (6) | 12.5% (7) | 76.8% (43) | 8 | **Strong consensus** |
| 29 | Since STP already has a pharmacokinetic interaction with CLB, no further dose reduction of CLB is necessary when adding CBD to a patient already receiving STP and CLB. | 20%  (11) | 21.8%  (12) | 58.2% (32) | 7 | **No Consensus** |
| 30 | When adding CBD to any base regimen of ASM including STP, the benefit of CBD should be assessed within 3 months. | 3.8%  (2) | 11.3%  (6) | 84.9% (45) | 8 | **Strong  consensus** |
| 31 | When adding CBD to a regimen containing STP, VPA, and CLB, if a partial seizure improvement and/or SE reduction was observed following STP addition and prior to CBD initiation, maintaining STP should be considered. | 0%  (0) | 5.7%  (3) | 94.3% (50) | 8 | **Strong  consensus** |
| 32 | When adding CBD to any base regimen of ASM including VPA, transaminase levels should be evaluated at baseline  and monitored during titration because of the potential for transaminitis in patients on combined VPA and CBD. | 0%  (0) | 0%  (0) | 100% (53) | 9 | **Strong  consensus** |
| 33 | When introducing CBD to a regimen containing STP, VPA, and CLB, most common adverse effects include sedation, somnolence, fatigue, ataxia, diarrhea, nausea, and anorexia. These are often transient, but if present, lower target CBD dose and slower CBD titration could be considered. | 0%  (0) | 5.7%  (3) | 94.3% (50) | 8 | **Strong  consensus** |
| **Patient follow-up during treatment initiation** | | | | | | |
| 34 | After adding a new treatment, an initial follow-up via a nurse and/or physician contact, telephone, or secure email within 1–2 weeks to assess new treatment’s safety and the family's confidence in the new ASM regimen may be considered. | 1.9%  (1) | 11.3%  (6) | 86.8% (46) | 9 | **Strong  consensus** |
| 35 | After adding a new treatment, a follow-up visit should be scheduled at 1–3 months post-initiation to assess safety and efficacy. | 0%  (0) | 0%  (0) | 100% (53) | 9 | **Strong  consensus** |
| 36 | When STP is added to CLB and VPA, blood counts and liver function should be assessed prior to starting treatment with STP and should be checked every 6 months unless otherwise clinically indicated. | 1.9% (1) | 7.5% (4) | 90.6% (48) | 8 | **Strong  consensus** |
| 37 | When FFA is added to any regimen, and to detect aortic or mitral valvular heart disease and pulmonary arterial hypertension, an echocardiogram should be performed at baseline and every 6 months after initiation for the first two years, and then eventually annually according to local regulation. Also, a final echocardiogram should be conducted 3-6 months after the last dose of treatment with FFA. | 0%  (0) | 1.9%  (1) | 98.1% (52) | 9 | **Strong  consensus** |
| 38 | When CBD is added to a regimen containing VPA, liver transaminases (AST, ALT) should be assessed at baseline  and at 6 weeks post-initiation due to the potential interaction between CBD and VPA, which may lead to elevated transaminase levels. A follow-up assessment of AST and ALT levels should also be conducted at 3 months. | 0% (0) | 3.8% (2) | 96.2% (51) | 9 | **Strong consensus** |

%1-2-3 represents the percentage of votes ≤3; n represents absolute number

%4-5-6 represents the percentage of votes ≥4 and ≤6; n represents absolute number

%7-8-9 represents the percentage of votes ≥7; n represents absolute number

**Supplementary Table S3:** List of voters agreeing to be acknowledged in the article (n=49/59)

| **Title** | **First name** | **Last name** | **Occupation** | **Affiliation** | **Country** |
| --- | --- | --- | --- | --- | --- |
| Dr. | Sergio | AGUILERA ALBESA | Pediatric Neurologist | Hospital Universitario de Navarra, Pamplona | Spain |
| Dr. | Maha | AWADALLA | Pediatric Neurologist | Royal London Hospital, London | United Kingdom |
| Dr. | Simona | BALESTRINI | Consultant Neurologist | Meyer Children’s Hospital IRCSS, University of Florence, University College of London, University College of London Hospitals | United Kingdom |
| Prof. | Immacolata Domenica | BATTAGLIA | Pediatric Neurologist | Policlinico Universitario Fondazione Agostino Gemelli, Roma | Italy |
| Dr. | Maria-Cristina | CAZACU | Pediatric Neurologist | Al.Obregia Hospital, Bucarest | Romania |
| Dr. | Elisabetta | CESARONI | Pediatric Neurologist | Salesi Hospital, Ancona | Italy |
| Dr. | Nicole | CHEMALY | Pediatric Neurologist | AP-HP Hôpital Necker Enfants Malades, Paris | France |
| Dr. | Catherine | CHIRON | Pediatric Neurologist | Retired | France |
| Dr. | Loucas | CHRISTODOULOU | Consultant Paediatrician with an interest in Paediatric Neurology | Chelsea & Westminster Hospital NHS Foundation Trust | United Kingdom |
| Prof. | Francesca | DARRA | Pediatric Neuropsychiatrist | Ospedale della Donna e del Bambino, Verona | Italy |
| Dr. | Archana | DESURKAR | Pediatric Neurologist | Sheffield Children’s Hospital | United Kingdom |
| Dr. | Anita | DEVLIN | Pediatric Neurologist | Great North Children’s Hospital, Newcastle | United Kingdom |
| Dr. | Pinelopi | DRAGOUMI | Pediatric Neurologist | Aristotle University of Thessaloniki, Thessalonique | Greece |
| Dr. | Ralf | EBERHARD | Paediatric epileptologist | Klinik Lengg, Zurich | Switzerland |
| Dr. | Maurizio | ELIA | Pediatric Neurologist | Ospedale IRCSS Oasi Maria, Troina | Italy |
| Dr. | Pi-Chuan | FAN | Physician | National Taiwan University Children’s Hospital, Taipei | Taïwan |
| Dr. | Alessandro | FERRETTI | Pediatric Neurologist | Sapienza University of Rome | Italy |
| Dr. | Santiago | FLESLER | Pediatric Neurologist | Hospital Alemán, Buenos Aires | Argentina |
| Dr. | Carmen | FONS | Pediatric Neurologist | Hospital Sant Joan de Déu Barcelona | Spain |
| Dr. | Ting-Rong | HSU | Physician | Taipei Veterans General Hospital | Taïwan |
| Dr | Salvador | IBÁÑEZ MICÓ | Pediatric Neurologist | Hospital Virgen de la Arrixaca, Murcia | Spain |
| Dr. | Naila | ISMAYILOVA | Pediatric Neurologist | Chelsea and Westminster Hospital NHS Foundation Trust | United Kingdom |
| Dr. | Nuria | LAMAGRANDE CASANOVA | Pediatric Neurologist | Hospital Infantil Universitario Niño Jesús, Madrid | Spain |
| Dr. | Julian | LARA-HERGUEDAS | Pediatric Neurologist | Hospital Universitario La Paz, Madrid | Spain |
| Dr. | Andrew | LUX | Pediatric Neurologist | Bristol Royal Hospital for Children | United Kingdom |
| Dr. | Sara | MATRICARDI | Pediatric Neurologist | University of Chieti-Pescara, Chieti | Italy |
| Dr. | Marina | NIKANOROVA | Pediatric Neurologist | University of Southern Denmark, Dianalund | Denmark |
| Prof. | Sylvie | NGUYEN THE TICH | Pediatric Neurologist | CHU Lille | France |
| Dr. | Katarína | OKÁĽOVÁ | Pediatric Neurologist | Detská fakultná nemocnica, Banská Bystrica | Slovakia |
| Prof. | Francesca | OPERTO | Pediatric Neurologist | University of Salerno | Italy |
| Prof. | Justyna | PAPROCKA | Pediatric Neurologist | Medical University of Silesia, Katowice | Poland |
| Dr. | Radu Stefan | PERJOC | Pediatric Neurologist | University of Medicine and Pharmacy, Bucharest | Romania |
| Dr. | Manish | PRASAD | Pediatric Neurologist | Queen’s Medical Centre, Nottingham | United Kingdom |
| Dr. | Antonella | RIVA | Pediatric Neurologist | Gaslini Hospital, Genova | Italy |
| Dr. | Celia | ROMERO DEL RINCÓN | Epileptologist | HU Vithas Madrid La Milagrosa, madrid | Spain |
| Dr. | Rocío | SÁNCHEZ-CARPINTERO ABAD | Pediatric Neurologist | Clínica Universidad de Navarra, Pamplona | Spain |
| Dr. | Irene | SÁNCHEZ-MIRANDA | Epileptologist | Hospital Ruber Internacional, Madrid | Spain |
| Dr. | Sílvia | SANCHEZ MARCO | Pediatric Neurologist | University Hospital of Wales, Cardiff | United Kingdom |
| Dr. | Benjamin | SERRAND | Pediatric Neurologist | CHU Rennes | France |
| Dr. | Annapurna | SUDARSANAM | Pediatric Neurologist | Birmingham Children’s Hospital | United Kingdom |
| Dr. | Nina | SWIDERSKA | Pediatric Neurologist | Royal Manchester Children’s Hospital | United Kingdom |
| Dr. | Calina | TODOSI | Pediatrician | CHRU Nancy | France |
| Dr. | Eulàlia | TURÓN-VIÑAS | Pediatric Neurologist | Hospital Sant Pau, Barcelona | Spain |
| Prof. | Kette | VALENTE | Pediatric Neurologist | Hospital das Clínicas da Faculdade de Medicina da Universidade de São Paulo | Brazil |
| Prof. | Patrick | VAN BOGAERT | Pediatric Neurologist | CHU Angers | France |
| Dr. | Maurizio | VIRI | Pediatric Neurologist | Maggiore Hospital, Novara | Italy |
| Prof. | Joanne | WILMSHURST | Pediatric Neurologist | Red Cross Children’s Hospital, Cape Town | South Africa |
| Dr. | Dimitrios | ZAFEIRIOU | Pediatric Neurologist | Aristotle University, Thessaloniki | Greece |
| Dr. | Marta | ZAWADZKA | Pediatric Neurologist | University Clinical Center, Gdansk | Poland |

Experts ordered alphabetically by last name.

**Supplementary Figure S1:** Clinical case #1 ”How to add STP on a base regimen containing DS non-specific ASMs?”

*STP: Stiripentol, VPA: valproate, CLB: Clobazam, DS: Dravet syndrome*

**

**Supplementary Figure S2:** Clinical case #2 ” How to add STP on a base regimen containing FFA?”

*STP: Stiripentol, VPA: valproate, CLB: Clobazam, FFA: Fenfluramine, DS: Dravet syndrome*

**

**Supplementary Figure S3:** Clinical case #3 ” How to add STP on a base regimen containing CBD?”

*STP: Stiripentol, VPA: valproate, CLB: Clobazam, CBD: Cannabidiol, LEV: Levetiracetam, DS: Dravet syndrome*

**

**Supplementary Figure S4:** Clinical case #4 ” How to add FFA on a base regimen containing STP?”

*STP: Stiripentol, VPA: valproate, CLB: Clobazam, FFA: Fenfluramine, DS: Dravet syndrome*

**

**Supplementary Figure S5:** Clinical case #5 ” How to add CBD on a base regimen containing STP?”

*STP: Stiripentol, VPA: valproate, CLB: Clobazam, CBD: Cannabidiol, DS: Dravet syndrome*

**
